# Targeted metabolomic analysis of exhaled breath condensate before and after bronchial provocation by eucapnic voluntary hyperventilation in adolescents with asthma: an exploratory study

**DOI:** 10.36416/1806-3756/e20250375

**Published:** 2026-08-13

**Authors:** Cláudio Gonçalves de Albuquerque, José Ângelo Rizzo, Edil de Albuquerque Rodrigues, Ricardo Oliveira Silva, Tatiane Priscila Santos Rodrigues da Luz, Marco Aurelio de Valois Correia, Décio Medeiros Peixoto

**Affiliations:** 1. Universidade Federal de Pernambuco, Recife (PE) Brasil.; 2. Laboratório de Metabonômica e Quimiometria. Departamento de Química Fundamental, Universidade Federal de Pernambuco, Recife (PE) Brasil.; 3. Programa de Pós-Graduação em Hebiatria e Programa de Pós-Graduação Associado em Educação Física, Faculdade de Ciências Médicas, Universidade Federal de Pernambuco, Recife (PE) Brasil.

**Keywords:** Asthma, Exercise-induced bronchospasm, Breath tests, Metabolomics

## Abstract

**Objective:**

To analyze endogenous metabolites in exhaled breath condensate before and after eucapnic voluntary hyperventilation (EVH) in adolescents with asthma with and without exercise-induced bronchoconstriction (EIB).

**Methods:**

This was an exploratory study including 44 adolescents with asthma in the 10- to 20-year age bracket. They were divided into two groups: the group of patients with EIB (n = 25) and that of those without (n = 19). EIB was defined as a ≥ 10% reduction in FEV_1_ after a six-minute EVH challenge using 5% carbon dioxide. Ventilation was maintained at 21 times the baseline FEV_1_. Exhaled breath condensate was collected before EVH and 15-30 min after EVH by using a validated device. Samples were analyzed by proton nuclear magnetic resonance focusing on the region of d1.60-2.00 parts per million. Data were preprocessed by using sum normalization and autoscaling for orthogonal partial least squares discriminant analysis.

**Results:**

All participants completed the protocol without adverse events. The mean reduction in FEV_1_ was significantly greater in the group of patients with EIB than in that of those without (−25.13% vs. −4.37%; mean difference, −20.76%; 95% CI, −26.45 to −15.07). In the EIB group, responses were classified as mild (n = 15), moderate (n = 9), or severe (n = 1). Post-EVH orthogonal partial least squares discriminant analysis showed significant separation between pre- and post-EVH metabolic profiles in the group of patients with EIB (p < 0.001) but not in that of those without (p = 0.195). The discriminative response in patients with EIB was associated with a marked increase in acetate signal intensity at 1.76 parts per million.

**Conclusions:**

Adolescents with EIB show a distinct metabolic fingerprint that is significantly associated with ventilatory stress. Acetate emerged as a key metabolic differentiator, suggesting a specific biochemical pathway associated with the exercise response and supporting its potential role in precision phenotyping of EIB.

## INTRODUCTION

Exercise-induced bronchoconstriction (EIB) is the narrowing of the lower airways during or after vigorous physical activity. It occurs in approximately 46% of young people with asthma, as well as in some individuals without asthma.^1^ ^,^ ^2^ The pathophysiology of EIB involves dehydration and cooling of the bronchial mucosa as a result of hyperventilation from intense exercise, triggering release of bronchoconstriction and inflammatory mediators.^2^ ^,^ ^3^

EIB is relevant because of its frequency and its impact on participation in sports and recreational activities, which are essential for quality of life and psychosocial development in young individuals.^4^ Despite advances in the understanding of EIB, it remains unclear why only some asthma patients develop EIB and why EIB may occur inconsistently within the same individual under similar exertion levels.^5^ ^,^ ^6^

Exhaled breath condensate (EBC) is a noninvasive biological sample that allows analysis of multiple chemical and biological components,^7^ ^,^ ^8^ contributing to a better understanding of the pathophysiology of asthma and specific phenotypes such as EIB. 

Metabolomics is a powerful tool to study the profile of small molecules, or metabolites, in biological samples. Some studies^9^ ^,^ ^10^ have successfully used this approach to distinguish individuals with asthma from those without through EBC analysis, underscoring its potential as a noninvasive tool to improve asthma diagnosis and phenotyping.^11^

We hypothesized that adolescents with asthma who develop EIB present with a unique metabolic fingerprint in EBC, which undergoes specific biochemical alterations triggered by eucapnic voluntary hyperventilation (EVH). 

The objective of the present proof-of-concept exploratory study was to compare the profiles of metabolites found in EBC obtained before and after bronchial provocation by EVH in adolescents with asthma with and without a response consistent with a diagnosis of EIB. 

## METHODS

This was a cross-sectional exploratory study conducted in the Pulmonary Function Laboratory of the Federal University of Pernambuco *Hospital das Clínicas*, located in the city of Recife, Brazil. The study was approved by the local research ethics committee (Protocol [CAAE] no. 91082318.7.0000.5208), and written informed consent was obtained from all participants and their legal guardians, in accordance with Brazilian legislation. Adolescents in the 10- to 20-year age bracket with a specialist-confirmed asthma diagnosis and referred for EIB testing were included. None were using controller medication, only a short-acting β_2_ agonist (albuterol) if needed. 

Patients were excluded if they were undergoing regular asthma control treatment or if they had a history of asthma worsening or upper airway infection symptoms in the previous four weeks. The exclusion of patients undergoing regular controller therapy was necessary to ensure that the observed metabolic profiles reflected the natural pathophysiology of EIB, thus avoiding potential confounding effects of anti-inflammatory medications. Patients with a baseline FEV_1_ below 60% of the predicted value were also excluded. 

Participant age was recorded, and height and weight were measured with calibrated equipment (Welmy, São Paulo, Brazil). On day one data on respiratory symptoms in the four weeks preceding the evaluation were collected, and spirometry with bronchodilator testing followed. Participants were asked to return after 48 h for bronchial challenge testing and EBC collection. 

### EBC collection procedures

**Figure 1:**
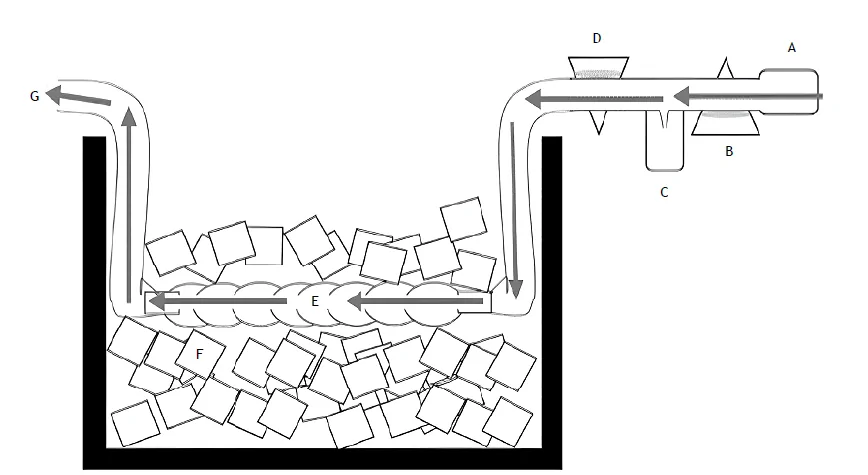
Diagram showing the equipment used in order to collect exhaled air condensate. In A, flexible silicone tube; in B, one-way valve; in C, mouthpiece for breathing; in D, one-way valve; in E, all submerged in water, cooking salt, and ice; in F, ice; and in G, air leaving the silicone tube.

EBC was collected for 15 min with a prototype of condensation device constructed and validated in our laboratory (see Discussion for a description of the validation process) and patented through the Brazilian National Institute for Industrial Property (Figure 1). 

During EBC collection, participants breathed tidally with their noses pinched through a mouthpiece and a one-way valve. Air was inhaled from the environment and exhaled through a silicone tube immersed in a temperature-controlled solution of water, sodium chloride, and ice, with the opposite end releasing air back. Pauses were allowed for swallowing saliva. Condensate was manually recovered from the tube and then stored at −80°C for metabolomic analysis in the Chemistry Department of the Federal University of Pernambuco. To ensure consistency, the condenser was maintained at a standardized mean temperature of 2.95 ± 1.35°C throughout all collections, yielding 5.26 ± 3.06 mL of biofluid. 

Component cleaning was standardized, and potential interference was assessed by performing metabolomic analysis of 10 samples of 5 mL of distilled water in silicone tubes after cleaning, showing no residues in proton nuclear magnetic resonance (NMR) spectra. Salivary contamination was minimized by using a unidirectional valve and was checked by comparing EBC spectra with previously published data on saliva spectra.^12^ Saliva-specific resonances (3-4 parts per million-ppm) were absent, thus confirming the absence of salivary contamination. 

Quality control procedures included standardized component cleaning, validation against reference samples, and monitoring for salivary contamination. These measures were implemented to ensure methodological consistency and reproducibility across all study participants. 

### Proton NMR-based metabolomic analysis

All proton NMR spectra were obtained by using a 400 MHz spectrometer (Varian Medical Systems, Palo Alto, CA, USA) at a proton frequency of 400 MHz. All frozen EBC samples were thawed at room temperature; prepared using 630 µL of sample and 70 µL of sodium phosphate buffer (0.2 M Na_2_HPO_4_ in 10% D_2_O, pH 7.0); and placed in 5 mm NMR tubes. One-dimensional NMR spectra were obtained by suppressing the water signal with the use of a standard pulse sequence (water suppression enhanced through T1 effects), with 14,522 data points in a spectral window of 5,186.7 Hz, an acquisition time of 1.4 s, a total of 160 transients, a saturation delay of 3.0 s, and a temperature of 22°C. The line broadening used was = 1 Hz. 

The signal attributed to the lactate methyl group (βCH_3_), i.e., δ1.33 ppm, was used as a chemical shift reference.^13^ ^,^ ^14^ The baselines were automatically corrected with MNova NMR software, version 12.0 (Mestrelab Research S.L.U., Santiago de Compostela, Spain) by using a Bernstein polynomial (full auto Bernstein polynomial fit). The phases of the spectra were corrected manually. In the present proof-of-concept study, we focused on a specific spectral signal (δ1.60-1.85 ppm) previously reported as being discriminative between individuals with asthma and those without.^9^ This targeted approach allowed us to model the metabolic response using a preselected marker with established predictive capacity rather than constructing a global metabolomic classification model. The region of the spectra between δ1.60 and 2.00 ppm was divided into regions of equal width of 0.004 ppm (bins),^15^ yielding a total of 67 bins to use the current peak as a target study.^9^ The evaluators responsible for spectral processing and statistical analysis were blinded to the clinical classification of participants (i.e., with EIB or without EIB). 

### EVH protocol

After 5-10 min, baseline FEV_1_ was measured and a six-minute bronchial provocation by EVH was subsequently carried out. FEV_1_ was measured again at 5, 10, 15, and 30 min after EVH, in accordance with American Thoracic Society/European Respiratory Society recommendations.^2^ ^,^ ^3^ EBC collection was performed again at 15-30 min after bronchial challenge testing. 

FEV_1_ was assessed with a microQuark spirometer (Cosmed, Rome, Italy), which was calibrated daily. FEV_1_ was defined as the highest value obtained from three acceptable maneuvers.^16^

EVH was performed in accordance with standardized protocols.^2^ ^,^ ^3^ Participants hyperventilated breathing a mixture of dry air containing 5% carbon dioxide to prevent respiratory alkalosis (White Martins, São Paulo, Brazil). The air was stored in appropriate cylinders and subsequently released into a series of Douglas bags and inhaled through a mouthpiece and a one-way low-resistance valve (Laerdal, Stavanger, Norway).

During EVH, participants maintained a target ventilation rate of 21 times the baseline FEV_1_ ^2^ ^,^ ^3^ for 6 min, measured with a spirometer (nSpire Health Inc., Waltham Abbey, UK). They received continuous encouragement, with brief pauses allowed for coughing or swallowing saliva. Safety criteria for interrupting procedures included an SpO_2_ of < 90%, marked distress, severe wheezing, or participant request, with albuterol available as rescue therapy. 

EIB was diagnosed when there was a decrease in FEV_1_ ≥ 10% in relation to the baseline at any post-EVH evaluation point. The reduction in FEV_1_ was classified as mild (> 10% and < 25%), moderate (≥ 25% and < 50%), or severe (> 50%).^2^ ^,^ ^3^

Environmental conditions in the air-conditioned laboratory were maintained at a mean temperature of 25.3 ± 1.56°C and a relative air humidity of 55.1 ± 3.3%. 

### Statistical analysis

The primary outcome was a change in metabolite signal intensity in the δ1.60-2.00 ppm spectral region before and after EVH. The main exposure variable was the presence of EIB, defined by an FEV_1_ reduction ≥ 10%. 

All clinical data were processed and analyzed with the IBM SPSS Statistics software package, version 20.0 (IBM Corporation, Armonk, NY, USA), and were entered by using double data entry. Any inconsistencies were investigated. The sample was characterized by using the Kolmogorov-Smirnov test for normality, and the Student’s t-test was used in order to compare independent continuous variables. 

A general matrix with 92 lines (cases) and 68 variables (bins of proton NMR spectra plus the class variable) was constructed, and multivariate analysis was carried out using the reference peak described elsewhere.^9^ All of the individuals in the matrix were asthma patients with or without EIB. The matrix was then divided into two: one for the participants with EIB, comparing samples collected before and after EVH; and one for those without EIB, also comparing samples collected before and after EVH. 

Intergroup comparisons of clinical and baseline data were performed with the Student’s t-test. Datasets were preprocessed with the use of sum normalization and autoscaling. Principal component analysis identified anomalous samples, and orthogonal partial least squares discriminant analysis created the two models via MetaboAnalyst 5.0 (Edmonton, Alberta, Canada). 

Sensitivity analyses were not conducted as part of the original study design; however, rigorous (leave-one-out) cross-validation and permutation testing were employed to ensure model robustness. 

## RESULTS

Forty-five adolescents were recruited, and one was excluded for having a baseline FEV_1_ below 60%. Among the final 44 participants, no EVH tests were interrupted because of severe respiratory complaints, and no adverse events or early interruptions occurred during the protocol. 

The sample was homogeneous in terms of age, weight, height, sex, and baseline FEV_1_, with no differences between individuals with and without EIB. Twenty-five (56.81%) of the 44 patients were found to have EIB after bronchial provocation by EVH (Table 1). Of the 25 adolescents with EIB, 15 (60%) were classified as showing mild FEV_1_ reduction after EVH, 9 (36%) were classified as showing moderate FEV_1_ reduction after EVH, and 1 (4%) was classified as showing severe FEV_1_ reduction after EVH. The mean difference in maximum FEV_1_ fall between groups was −20.76% (95% CI, −26.45 to −15.07; p < 0.001). 

**Table 1:** Clinical characteristics and stratification of exercise-induced bronchoconstriction severity among the study participants.^a^

Group	EIB (n = 25/56.8%)	Without EIB (n = 19/43.2%)	p*
Age, years	12.7 ± 3.0	15.0 ± 3.2	0.74
Weight, kg	46.8 ± 14.4	63.8 ± 21.8	0.54
Height, cm	151.4 ± 14.0	153.79 ± 39.9	0.73
Male sex	12 (52.2%)	11 (57.9%)	0.45
FEV_1_, L/s	2.38 ± 0.7	3.33 ± 0.9	0.77
FEV_1_, % predicted	91.6 ± 15.3	99.0 ± 15.9	0.73
Maximum FEV_1_ fall after EVH, %	−25.13 ± 14.9	−4.37 ± 3.6	< 0.001
EIB severity			
Mild (> 10% and < 25% fall)	15 (60%)		
Moderate (≥ 25% and < 50% fall)	9 (36%)		
Severe (> 50% fall)	1 (4%)		

EIB: exercise-induced bronchoconstriction; and EVH: eucapnic voluntary hyperventilation. ^a^Data expressed as n (%) or mean ± SD. *The Student’s t-test was used for intergroup comparisons.

**Figure 2:**
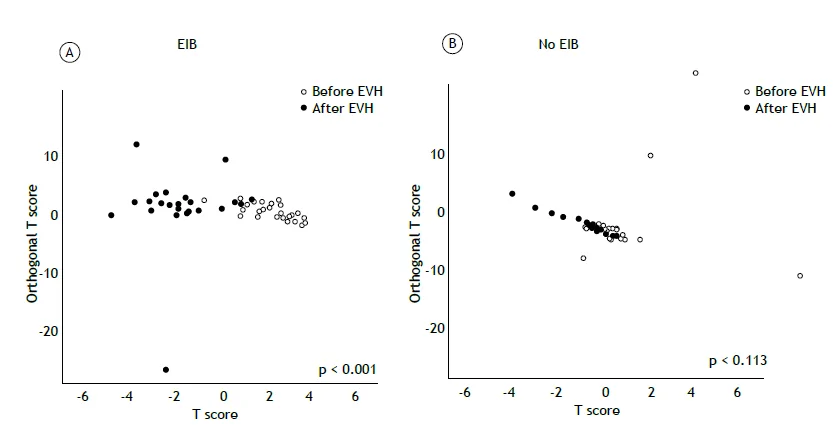
Score plot of orthogonal partial least squares discriminant analysis of comparison of the profile of metabolites in exhaled breath condensate (EBC) before and after eucapnic voluntary hyperventilation (EVH) in adolescents with asthma with and without exercise-induced bronchoconstriction (EIB). Note that the line in the center shows separation of groups, a mathematical representation of proton nuclear magnetic resonance spectrum signals of metabolites that best distinguish metabolites in EBC before and after EVH.

Regarding the metabolomic analysis, orthogonal partial least squares discriminant analysis was used in order to compare EBC metabolite profiles before and after the EVH challenge. In adolescents with EIB, a significant discriminative separation was observed between pre- and post-challenge samples (p < 0.001; Figure 2A). In contrast, no significant metabolic alterations were detected in the group of asthma patients without EIB (p = 0.195; Figure 2B). 

Model performance metrics for the EIB group indicated modest discrimination, with moderate explanatory capacity (R^2^ = 0.332) and limited-to-moderate predictive ability (Q^2^ = 0.226). Leave-one-out cross-validation and 2,000-permutation testing confirmed statistical significance (p < 0.001). In contrast, the model for patients without EIB showed substantially lower performance (R^2^ = 0.273; Q^2^ = 0.0962; p = 0.195), indicating no significant metabolic alterations in asthma patients without EIB. The positive Q^2^ value (0.226) in the EIB model confirmed the predictive validity of the discriminant analysis, whereas the Q^2^ in the model for patients without EIB (0.0962) further supported the absence of metabolic response in this group. 

The validity of these discriminative models was further supported by the 2,000-permutation test, which confirmed that the observed differences in the EIB group were statistically significant and not due to chance (p < 0.001), whereas the model for individuals without EIB failed to show significant discrimination (p = 0.195). 

Two score graphs were generated by using the two models (Figure 2): one for the EIB-positive model (Figure 2A) and one for the EIB-negative model (Figure 2B). Each circle in the graph represents a sample of EBC before (open circles) and after (solid circles) application of the EVH test. A shorter distance between samples indicates similar EBC metabolite profiles, whereas greater distances indicate differences. 

**Figure 3:**
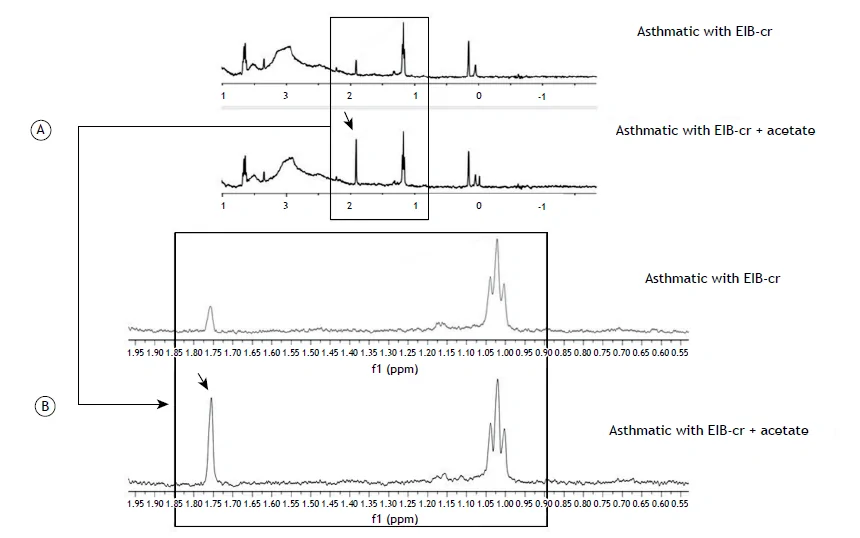
Detail of proton nuclear magnetic resonance spectra of a random sample from the group of asthma patients with exercise-induced bronchoconstriction (EIB) before (in A) and after (in B) addition of dilute acetic acid solution to compare increases in acetate signal intensity at δ1.76 parts per million (ppm).

The discriminative metabolic response in the EIB group was primarily associated with a significant increase in the proton NMR signal intensity at 1.76 ppm, which was identified as acetate. This association was subsequently confirmed in the laboratory by adding a dilute solution of acetate to the sample (Figure 3). 

**Figure 4:**
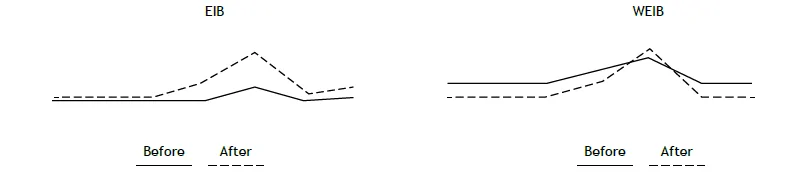
Average proton nuclear magnetic resonance spectra of exhaled breath condensate in the region of δ1.60-1.85 parts per million. In A, adolescents with asthma and exercise-induced bronchoconstriction (EIB) showed a clear change after eucapnic voluntary hyperventilation when compared with baseline. In B, no relevant changes were observed in adolescents with asthma without EIB before and after the challenge.

The average spectra obtained from the metabolomic models in the δ1.60-1.85 ppm region are shown in Figure 4. Among the adolescents with asthma and EIB, a clear change was observed after bronchial provocation when compared with baseline (Figure 4A). In contrast, no relevant change was detected in the spectra for adolescents with asthma without EIB before and after the challenge (Figure 4B). These findings reinforce that the metabolic response in this spectral region occurs only in individuals who develop EIB. 

## DISCUSSION

Our results show a difference in the region of the proton NMR spectrum under analysis in the EBC of adolescents with asthma and EIB before and after bronchial provocation by EVH. This change was not found in asthma patients without EIB. Spectrum analysis identified acetate as the metabolite associated with the change in the metabolic profile of these individuals with EIB. 

EBC is a noninvasive sample that is used in order to detect biomarkers such as cytokines and eicosanoids, reflecting airway inflammation.^17^ ^,^ ^18^ Studies using proton NMR have analyzed EBC metabolites in asthma,^16^ ^,^ ^19^ ^,^ ^20^ with additional use of spectrometry to assess markers such as cysteinyl leukotrienes (CysLTs). One study^21^ found increased CysLT levels in children with EIB, supporting their role in EIB. 

Recent research indicates that EBC pH is sensitive for predicting asthma in children,^22^ whereas inhaled corticosteroids reduce CysLT levels. Studies with proton NMR have identified spectral differences that distinguish individuals with asthma from those without and allow subcategorization based on eosinophilia and corticosteroid use.^19^

Our EBC collection equipment was validated by analyzing the metabolic profiles of 47 adolescents, of whom 12 were individuals without asthma or respiratory symptoms and 35 were individuals with asthma. Proton NMR metabolomics accurately distinguished between the two groups (89.8%), with a sensitivity of 94.6% and a specificity of 75.0%, thus confirming the robustness of the model. This discriminative capacity is consistent with previous studies,^9^ ^,^ ^10^ further validating our methodology for distinguishing individuals with asthma from those without. 

It is important to note that EBC composition is sensitive to methodological variations, and differences in condensation systems/temperature may affect metabolite detection and reproducibility. Thus, our findings should be interpreted within this context and require external validation. 

Further analyses identified metabolites such as succinate, pyruvate, and saturated fatty acids as markers distinguishing asthma patients from control individuals,^23^ reinforcing the relevance of EBC in studying inflammation and evaluating respiratory treatment. 

One study^16^ used proton NMR to analyze EBC from 25 children with controlled allergic asthma and 11 healthy controls, achieving up to 86% accuracy in spectral discrimination. Acetylated compounds were identified in the 1.7-2.2 ppm region, with differences near 1.86-1.92 ppm in adolescents with EIB before and after EVH. Our study identified an acetate signal in asthma patients who developed EIB, suggesting activation of a specific metabolic pathway. This finding indicates a distinct biochemical signature and a targeted metabolic response to ventilatory stress. The absence of metabolic changes after bronchial provocation in asthma patients without EIB suggests that responses to stimuli such as hyperventilation are specifically modulated in EIB. Our data support that although EIB and asthma are related, they have distinct pathophysiological mechanisms. 

Acetate has been linked to inflammatory pathways in asthma and exercise, modulating airway contraction, protein acetylation, and immune activation, thus reinforcing its role in acute inflammatory responses.^14^ ^,^ ^16^ ^,^ ^24^ This metabolite can be generated through degradation of pyruvate and butyrate, both of which have been described as modulators of inflammation and airway smooth muscle contraction.^9^ ^,^ ^25^ ^,^ ^26^

Although acetate has previously been associated with metabolic and inflammatory pathways, its mechanistic interpretation in EIB should be approached with caution. The present findings show an association between increased acetate signal intensity and EIB following ventilatory stress; however, causal relationships and underlying biological mechanisms cannot be definitively established within the scope of this exploratory study design. 

Higher acetate levels in EBC may promote protein acetylation, including high mobility group box 1, leading to its translocation from the nucleus to the cytoplasm and subsequent extracellular release. This process intensifies inflammation by activating immune cells and stimulating cytokine production.^27^ ^,^ ^28^ Thus, acetate acts not only as a metabolic marker but also as a potential modulator of airway inflammation, especially under metabolic stress. 

**Figure 5.Schematic:**
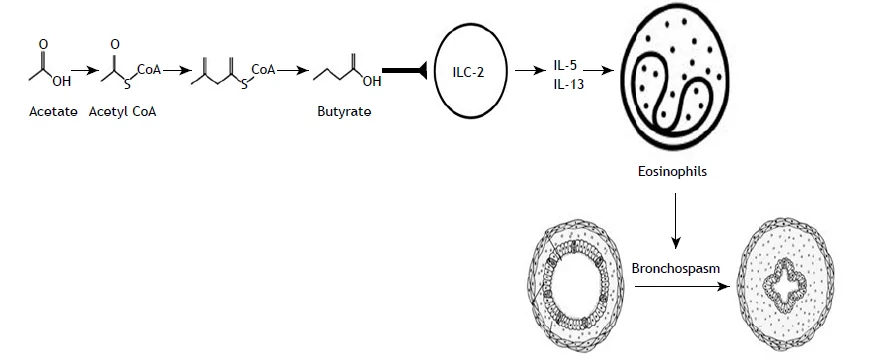
illustration showing acetate metabolic pathways during exercise-induced bronchoconstriction. *Adapted from Chang-Chien et al.^9^ CoA: coenzyme A; and ILC2s: group 2 innate lymphoid cells.

In the proposed model (Figure 5), acetate plays a key role in activating group 2 innate lymphoid cells (ILC2s). Recent studies have shown that ILC2 activation by mediators such as thymic stromal lymphopoietin and IL-33, combined with CysLTs, amplifies the type 2 inflammatory response. One study^28^ demonstrated that leukotriene C4 enhances IL-33-mediated ILC2 activation, increasing production of Th2 cytokines (IL-5 and IL-13), as well as promoting cell proliferation and eosinophil accumulation in the lungs. These findings highlight leukotriene C4 as an important modulator of receptors such as cysteinyl leukotriene receptor 1 in ILC2s, establishing an autocrine cycle that exacerbates lung inflammation, central to EIB. 

Activation of ILC2s by leukotrienes such as leukotriene D4 via cysteinyl leukotriene receptor 1 highlights their role in EIB pathophysiology. One study^29^ ^,^ ^30^ showed that leukotriene D4 induces IL-4, IL-5, and IL-13 production by ILC2s and promotes their proliferation and accumulation, effects that are reversible by cysteinyl leukotriene receptor 1 antagonism. These mechanisms illustrate the interaction between lipid mediators and cytokines in the lung microenvironment, suggesting that acetate may serve as a metabolic precursor in CysLT production, thereby amplifying inflammatory responses in EIB. 

Although American Thoracic Society/European Respiratory Society guidelines recommend standardized collection systems,^31^ ^,^ ^32^ our proof-of-concept study used a laboratory-developed device because of cost and limited availability. Although this limits direct comparability with standard methods, the device was internally validated and was operated under controlled conditions, including standardized condenser temperature, environmental monitoring, and strict cleaning protocols. These measures ensured sufficient consistency to detect group differences and identify acetate, demonstrating the feasibility of EBC metabolomic analysis in resource-limited settings. Despite not fully conforming to international standards, the custom device was adequate for the exploratory aims of the present study. 

The relatively small sample size may limit statistical power and increase the risk of type II error, particularly in detecting subtle metabolic differences in the group of patients without EIB. Although standardization procedures were applied, residual variability inherent to EBC metabolomics cannot be fully excluded. The collection tube temperature (2.95 ± 1.35°C), despite being standardized across participants, may have limited the detection of certain metabolites, and the use of a 400 MHz NMR system, in comparison with higher-field instruments (800-1,200 MHz), may also have reduced analytical sensitivity. However, these limitations are expected and appropriate for a proof-of-concept exploratory study designed to generate hypotheses rather than provide definitive conclusions. 

The present study suggests promising ways toward a better understanding of the mechanisms that trigger EIB in individuals with asthma. Investigation of these mechanisms may help to develop more effective strategies for prevention and management of EIB during and after physical exercise. 
